# Mechanisms and outcomes of flipped music education: a conceptual framework linking self-directed learning, collaboration, and student wellbeing

**DOI:** 10.3389/fpsyg.2026.1892285

**Published:** 2026-07-23

**Authors:** Qisen Zhu, Jinglong Li

**Affiliations:** 1 Faculty of Education, University Kebangsaan Malaysia, Selangor, Malaysia; 2 Industrial Design College, LuXun Academy of Fine Arts, Shenyang, China

**Keywords:** collaborative learning, emotional regulation, flipped music education, learning engagement, self-directed learning, student wellbeing

## Abstract

Flipped music education has increasingly emerged as a learner-centered instructional approach that promotes active participation, self-regulated learning, and collaborative engagement. In music theory education, students often encounter difficulties in understanding abstract concepts and connecting theoretical knowledge with practical application. Although flipped learning has shown positive educational effects, existing evidence remains fragmented and primarily emphasizes instructional implementation rather than the mechanisms underlying broader developmental outcomes. This study therefore synthesizes current evidence concerning how flipped music education influences student engagement, learning experiences, and wellbeing while proposing an integrated conceptual framework. A systematic literature review following the PRISMA 2020 guidelines was conducted using Scopus, Web of Science, and ERIC databases. Following a multi-stage screening process, 12 peer-reviewed studies published between 2015 and 2025 were included in the final synthesis. The findings indicate that instructional design, collaborative participation, teacher support, assessment practices, and self-regulated learning collectively contribute to cognitive engagement, conceptual understanding, motivation, social connectedness, and wellbeing-related outcomes. Based on these findings, the study proposes a conceptual framework explaining how instructional and contextual factors indirectly influence student development through engagement, collaboration, and reflective learning processes. The review contributes to current scholarship by advancing a mechanism-oriented understanding of flipped music education and providing practical implications for developing more engaging and supportive music learning environments.

## 1 Introduction

Music education has increasingly been recognized as an educational domain extending beyond the acquisition of musical knowledge and skills to broader cognitive, emotional, and developmental dimensions (Váradi, 2022). Contemporary educational research suggests that engagement in musical activities can facilitate active learning processes, strengthen social interaction, and contribute to students' overall educational experiences (Bussu and Mangiarulo, 2024). As educational systems continue to emphasize learner-centered practices and holistic development, increasing attention has been directed toward instructional approaches capable of promoting autonomy, engagement, and meaningful knowledge construction within music learning environments (Wang et al., 2025).

Despite these developments, traditional approaches to music theory instruction often remain predominantly teacher-centered, relying on lectures and repetitive exercises for knowledge transmission (Ge et al., 2025). Although such methods may facilitate the acquisition of fundamental concepts, they frequently provide limited opportunities for active participation and individualized learning (Brown and Palincsar, 2018). Music theory education commonly involves abstract constructs, including harmonic organization and formal analysis, which require continuous interaction between conceptual understanding and practical application (Utne-Reitan, 2022). Consequently, students may experience difficulties in sustaining motivation and connecting theoretical knowledge with authentic learning contexts (Roach et al., 2018). Understanding students' learning needs has therefore become an essential prerequisite for designing effective flipped music learning environments that can accommodate diverse learner characteristics and support meaningful educational experiences.

Within this context, the flipped classroom model has emerged as an increasingly influential pedagogical approach because of its potential to transform passive learning environments into more active and participatory experiences (Jiang et al., 2022). By relocating foundational knowledge acquisition to pre-class activities and reserving classroom time for discussion, collaborative engagement, and applied learning tasks, flipped learning enables students to participate more actively in higher-order learning processes (Hwang et al., 2019). Existing studies indicate that this instructional approach may enhance learner engagement, strengthen classroom participation, and provide greater flexibility in learning experiences (He, 2020).

Nevertheless, the effectiveness of flipped learning extends beyond modifications in instructional structure and may depend on multiple interconnected learning mechanisms operating within these environments (Uddin and Mc Neill, 2024). Previous studies suggest that learning needs, self-directed learning, collaborative learning, curriculum design, and assessment practices collectively shape students' learning experiences and influence broader educational outcomes. Among these mechanisms, self-directed learning plays a particularly important role in supporting successful participation in flipped classrooms (Brame, 2017; Awidi and Paynter, 2019; Qisen et al., 2024). During pre-class learning activities, students assume greater responsibility for regulating learning behaviors, establishing learning goals, and managing independent learning tasks (Zheng et al., 2020). At the same time, collaborative interactions within classroom settings may further support knowledge construction through discussion, peer engagement, and shared learning experiences (Zheng et al., 2023; Sartania et al., 2022). Together, these processes may contribute not only to learning effectiveness but also to broader educational outcomes associated with engagement and wellbeing.

Although positive outcomes of flipped learning in music education have been documented, current evidence remains relatively fragmented and predominantly focuses on instructional implementation and academic performance. Less attention has been directed toward understanding how interconnected learning processes—including learning needs self-directed learning, collaborative engagement, curriculum design, and assessment mechanisms—collectively influence broader educational experiences and developmental outcomes. Consequently, a coherent framework explaining the pathways through which flipped music education contributes to learning engagement and student wellbeing remains insufficiently developed.

To address this limitation, the present study employs a systematic literature review to synthesize existing evidence and develop a conceptual framework for understanding learning needs and instructional mechanisms in flipped music education. By synthesizing theoretical perspectives and empirical evidence, this study seeks to clarify the relationships among learning needs, self-directed learning, collaborative learning, curriculum design, and assessment mechanisms within flipped learning environments. Rather than conceptualizing these dimensions as independent instructional elements, the proposed framework positions them as interconnected mechanisms that shape engagement, emotional experiences, and broader educational outcomes.

The proposed framework contributes both theoretically and practically. Theoretically, it advances understanding of the mechanisms underlying flipped music education and extends current discussions concerning instructional innovation in music learning contexts. Practically, it offers educators a structured perspective for improving instructional design and supporting meaningful student participation. Accordingly, a preliminary conceptual framework is proposed to explain how learning needs interact with curriculum design, self-directed learning, collaborative learning, and assessment practices to influence student engagement, emotional regulation, wellbeing, and overall learning outcomes in flipped music education.

## 2 Methodology

### 2.1 Research question

This systematic review aims to examine how flipped music education influences learning processes and broader educational outcomes by synthesizing empirical evidence published between 2015 and 2025. The review was limited to this period because research on flipped music education expanded substantially after 2015, reflecting the increasing integration of digital learning technologies, learning management systems, and learner-centered pedagogies in music education. Earlier studies were relatively sparse and largely exploratory, whereas research published since 2015 provides a more robust evidence base for examining instructional mechanisms, student engagement, and wellbeing outcomes. Consequently, this timeframe was considered appropriate for capturing the current state of knowledge while ensuring the relevance of the proposed conceptual framework.

### 2.2 To address this objective, the following research questions (RQs) were formulated

**RQ1:** What instructional and contextual factors support effective flipped music learning environments?

**RQ2:** How do these factors influence students' engagement and learning experiences?

**RQ3:** What cognitive, emotional, and wellbeing outcomes are associated with flipped music education?

**RQ4:** How can existing evidence be synthesized to explain the mechanisms through which flipped music education contributes to student development and wellbeing?

This study employed a systematic literature review approach to identify and synthesize relevant studies concerning flipped music education and its associated learning processes and outcomes. Literature searches were conducted across major academic databases, including Scopus, Web of Science (WoS), and ERIC, to ensure comprehensive coverage of relevant educational and interdisciplinary research. The search strategy was developed using a combination of terms related to instructional approaches, educational contexts, and learning outcomes, including: *(“flipped classroom” OR “flipped learning” OR “inverted classroom”) AND (“music education” OR “music learning” OR “music teaching” OR “music theory” OR music) AND (learning OR engagement OR motivation OR wellbeing OR cognition OR achievement OR student)*. The search was limited to studies published between 2015 and 2025, reflecting recent developments in flipped learning and music education research. Only English-language publications were included to ensure consistent interpretation of study methods and findings during the review process. Although AI-assisted translation has improved accessibility to non-English literature, this criterion may have introduced language bias and is acknowledged as a limitation of the review. Following the initial database search, retrieved records were screened and evaluated according to predefined inclusion criteria. As presented in Table 1, the search process initially identified a total of 151 articles for further review and selection.

**Table 1:** Web of science, scopus, and ERIC keyword.

Journal search	Search content	Results
Web of science	TS= ((“flipped classroom” OR “flipped learning” OR “inverted classroom”) AND (“music education” OR “music learning” OR “music teaching” OR “music theory” OR music) AND (learning OR engagement OR motivation OR wellbeing OR cognition OR achievement OR student))	51
Scopus	TITLE-ABS-KEY ((“flipped classroom” OR “flipped learning” OR “inverted classroom”) AND (“music education” OR “music learning” OR “music teaching” OR “music theory” OR music) AND (learning OR engagement OR motivation OR wellbeing OR cognition OR achievement OR student)) AND PUBYEAR > 2015 AND PUBYEAR < 2025	70
ERIC	((“flipped classroom” OR “flipped learning” OR “inverted classroom”) AND (“music education” OR “music learning” OR “music teaching” OR “music theory” OR music) AND (learning OR engagement OR motivation OR wellbeing OR cognition OR achievement OR student))	30

This study aims to develop a conceptual understanding of flipped music education by systematically synthesizing existing evidence regarding its underlying learning mechanisms and educational outcomes. Specifically, the review focuses on the relationships among self-directed learning, collaborative learning processes, and broader outcomes associated with student engagement and wellbeing. A systematic review methodology was adopted to ensure a structured and transparent process of evidence identification and selection. As presented in Table 2, the review procedure consisted of sequential stages, including identification, screening, eligibility assessment, inclusion, and exclusion, to ensure the relevance and quality of the selected studies.

**Table 2:** Inclusion and exclusion criteria for publications.

Inclusion criteria	Exclusion criteria
Published between 2015–2025	Published before 2015
Indexed in Scopus, WoS, and ERIC databases	Published in databases outside Scopus, WoS, and ERIC
Published in peer-reviewed journals	Non-peer-reviewed publications
Full-text articles available	Unavailable full-text articles
Studies focusing on flipped learning in music education contexts	Studies unrelated to music education
Studies examining learning processes (e.g., self-directed learning, collaborative learning, engagement)	Studies unrelated to learning mechanisms
Studies reporting educational outcomes (e.g., learning outcomes, wellbeing, cognition, engagement)	Studies unrelated to educational outcomes

This systematic literature review followed the PRISMA 2020 (Preferred Reporting Items for Systematic Reviews and Meta-Analyses) framework to ensure transparency, reproducibility, and methodological rigor throughout the identification, screening, eligibility assessment, and study selection processes. The review aimed to synthesize empirical evidence concerning flipped music education, with particular emphasis on instructional design, student engagement, self-directed learning, collaborative learning, and educational wellbeing.

Studies were retrieved from three major electronic databases, namely Web of Science, Scopus, and ERIC, which were selected because of their broad coverage of peer-reviewed research in education, instructional technology, and music education. The search was limited to publications published between January 2015 and December 2025, reflecting a period during which technology-enhanced learning and flipped instructional approaches became increasingly prevalent in higher education.

The database search identified 151 records, comprising 70 records from Scopus, 51 from Web of Science, and 30 from ERIC. All references were imported into Zotero for reference management and duplicate detection. After 32 duplicate records were removed, 119 unique studies remained for further screening.

Title screening was conducted to evaluate the relevance of each study to flipped music education and the objectives of the review. This process resulted in the exclusion of 48 records that did not demonstrate sufficient topical relevance, leaving 71 studies for abstract screening. Abstracts were subsequently examined to determine whether the studies addressed flipped music education and relevant learning processes. Following this assessment, 35 studies were excluded because they did not adequately correspond with the review objectives, resulting in 36 articles being retained for full-text evaluation.

Full-text articles were assessed against the predefined inclusion and exclusion criteria. A total of 24 studies were excluded during this stage. The excluded studies comprised those unrelated to flipped music education (*n* = 7), studies that did not address self-directed learning or collaborative learning processes (*n* = 4), studies lacking evidence related to learning engagement, emotional regulation, or student wellbeing (*n* = 3), studies providing insufficient methodological information or incomplete reporting (*n* = 2), non-peer-reviewed publications such as conference proceedings (*n* = 3), gray literature including theses, dissertations, books, and government reports (*n* = 2), full-text articles that were unavailable or inaccessible (*n* = 1), and publications written in languages other than English (*n* = 2).

**Figure 1:**
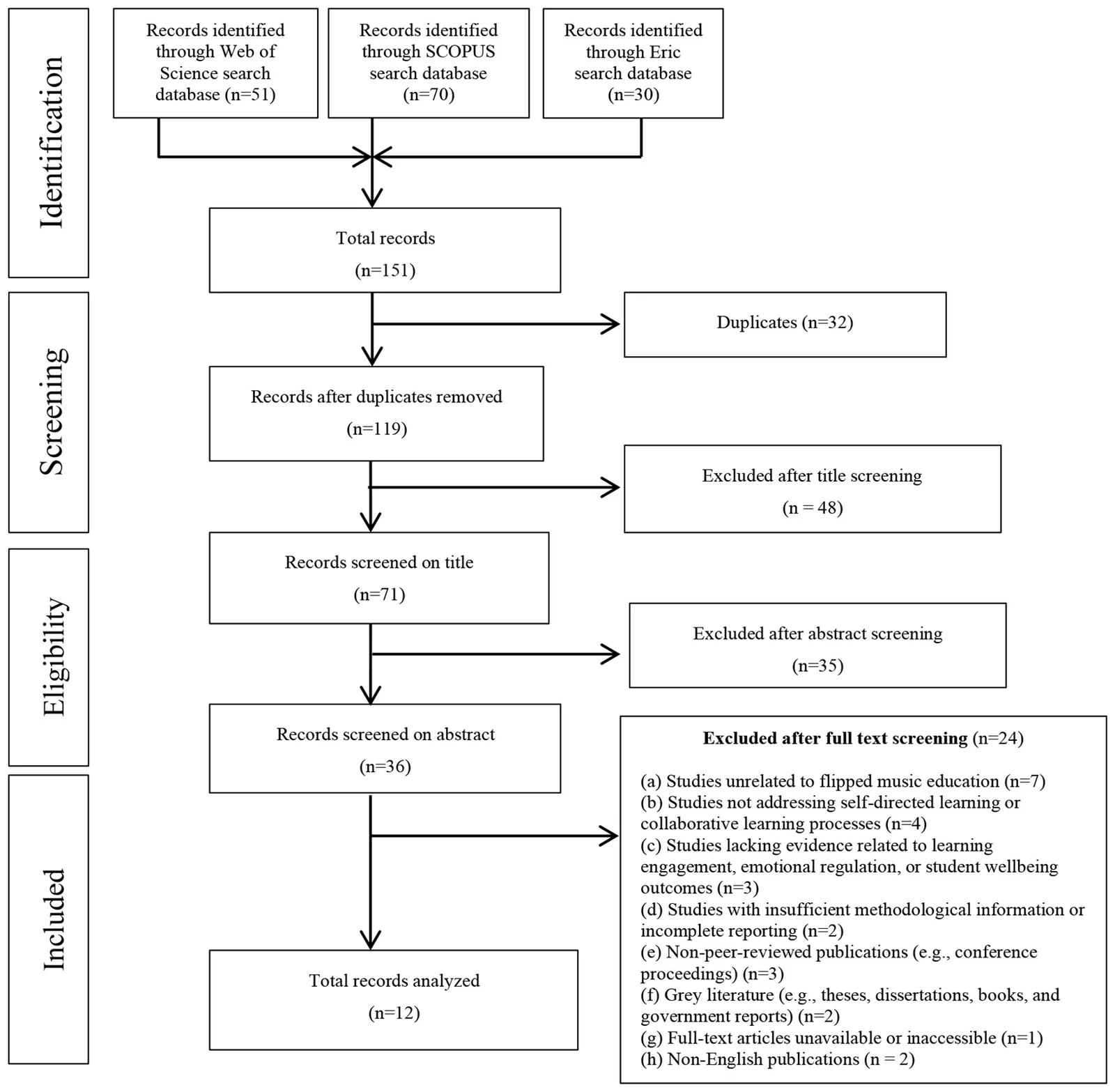
PRISMA diagram for the systematic review (Page et al., 2021).

Application of these eligibility criteria ensured that only methodologically robust studies with direct relevance to the review objectives were retained. Consequently, 12 empirical studies satisfied all inclusion criteria and were included in the final qualitative synthesis. Any disagreements arising during the screening process were resolved through discussion and consensus among the reviewers to enhance consistency and minimize potential selection bias. The complete study selection process is presented in Figure 1.

### 2.3 Data extraction

Following study selection, a structured data extraction procedure was conducted to ensure consistent organization of the included studies. For each of the 12 selected articles, information relating to authorship, publication year, research objectives, participant characteristics, methodological design, instructional context, intervention characteristics, and key findings was systematically recorded.

To examine the learning mechanisms underlying flipped music education, both deductive and inductive coding strategies were employed. Deductive coding was guided by the review focus on self-directed learning, collaborative learning, student engagement, and wellbeing outcomes, whereas inductive coding enabled additional patterns and contextual factors to emerge from the literature. Data organization and coding were conducted using Microsoft Excel. To enhance analytical consistency, two reviewers independently coded the studies and resolved discrepancies through discussion and consensus. This process established a structured basis for identifying relationships among learning processes and educational outcomes within flipped music education contexts.

### 2.4 Data synthesis

Given the methodological heterogeneity of the included studies, a quantitative meta-analysis was not undertaken. Variations in research design, participant characteristics, instructional contexts, and outcome measures limited the comparability required for statistical aggregation. Therefore, a narrative and thematic synthesis approach was adopted, consistent with recommendations for reviews involving diverse educational evidence (Siddaway et al., 2019).

The synthesis proceeded in two stages. First, descriptive mapping summarized the characteristics of the included studies, including research design, participant profiles, and instructional settings. Second, thematic synthesis identified recurring patterns and relationships among self-directed learning, collaborative learning, student engagement, and wellbeing outcomes. Through iterative comparison across studies, these findings were integrated to support the development of a conceptual framework for flipped music education.

## 3 Results

A total of 12 empirical studies met the inclusion criteria and were included in the final synthesis. These studies were published between 2016 and 2025 and represented diverse geographical contexts, including China, Hong Kong, Japan, Australia, Greece, Spain, and Türkiye. Methodologically, the evidence comprised randomized controlled trials, quasi-experimental studies, mixed-methods research, qualitative case studies, and descriptive case studies, reflecting considerable methodological diversity. Participants ranged from secondary school and undergraduate music students to music teachers, teacher candidates, novice piano learners, and family members, indicating that flipped music education has been implemented across multiple educational contexts. Despite differences in research design and participant characteristics, the included studies consistently reported positive educational effects related to learner engagement, musical competence, motivation, collaboration, self-directed learning, and learning performance. The characteristics of the included studies are presented in Table 3.

**Table 3:** Characteristics of the included studies.

References	Country of origin	Methodology	Participants	Findings	Implications
Lv (2023)	China	Randomized controlled trial (RCT)	118 novice piano students	Flipped learning significantly improved piano performance, self-regulated learning, and engagement compared with traditional instruction.	Flipped learning is an effective instructional strategy for beginner piano education and supports autonomous learning.
Ng et al. (2022)	Hong Kong	Mixed-methods case study	122 secondary school students	Online flipped learning enhanced learning motivation, collaboration, and digital competence.	Technology-supported flipped learning can facilitate active music learning in secondary education.
Roig-Vila and Fabra-Brell (2024)	Spain	Mixed-method collective case study	25 students; 13 family members	Students and families showed high acceptance of flipped learning; autonomy and engagement increased.	Family involvement and equitable technology access are important for sustainable implementation.
Perakaki (2025)	Greece	Qualitative case study	Two music teachers	Flipped learning promoted active classroom interaction but required extensive preparation time.	Teacher training and curriculum support are essential for successful adoption.
Sakin et al. (2024)	Turkey	Explanatory sequential mixed methods	Music education students	Students reported improved achievement and positive learning experiences.	Combining quantitative and qualitative evidence provides a comprehensive evaluation of flipped learning.
Ozdamar and Yavuz-Konokman (2024)	Turkey	Qualitative case study	Music teacher candidates	Flipped learning increased participation, critical thinking, and collaboration.	Active learning environments should be integrated into teacher education.
Zhu et al. (2025)	China	Quasi-experimental study	60 undergraduate students	Flipped Classroom Module significantly improved music theory knowledge and musical competence.	Well-designed flipped modules enhance undergraduate music education outcomes.
Brell and Roig-Vila (2022)	Spain	Mixed-method case study	Secondary music students	Students perceived greater flexibility and engagement using flipped learning.	Digital readiness and instructional design influence learning effectiveness.
Yilmaz and Zahal (2023)	Turkey	Randomized pretest-posttest experiment	Undergraduate music students	Experimental group achieved significantly higher academic performance.	Flipped learning can outperform conventional instruction in higher music education.
Doi (2016)	Japan	Flipped classroom implementation	First-year undergraduate music students	Students demonstrated stronger preparation and participation in class activities.	Early adoption of flipped learning encourages learner responsibility.
Nethsinghe et al. (2023)	Australia	Descriptive case study	Music education students	Online flipped learning-maintained continuity of music education during COVID-19.	Blended and online pedagogies increase resilience in music education.
Fan (2025)	China	Controlled pretest-posttest experiment	University music students	AI-assisted flipped learning significantly enhanced musical creativity and learning performance.	Integrating AI with flipped learning represents a promising direction for music education.

### 3.1 RQ1: what instructional and contextual factors support effective flipped music learning environments?

The synthesis of the included studies indicates that effective flipped music learning environments are supported by the coordinated integration of instructional design, learner-centered pedagogy, teacher facilitation, collaborative learning, and formative assessment. Rather than functioning as discrete instructional components, these pedagogical and contextual factors operate as an interconnected instructional system that enables students to prepare independently, participate actively during classroom learning, and continuously regulate their learning throughout the instructional process. Across the reviewed studies, successful flipped music instruction consistently followed a coherent learning sequence in which pre-class knowledge acquisition was integrated with interactive classroom learning and reflective post-class consolidation, thereby creating conditions that support meaningful musical learning and sustained learner engagement (Perakaki, 2025; Roig-Vila and Fabra-Brell, 2024; Zhu et al., 2025).

Instructional design emerged as the primary pedagogical foundation underpinning flipped music learning. Across the reviewed studies, students engaged with instructional videos, digital learning resources, guided learning materials, AI-supported platforms, and music theory exercises before attending class. This pre-class preparation enabled learners to acquire foundational knowledge at their own pace, revisit challenging musical concepts when necessary, and arrive in class with greater conceptual readiness. Consequently, classroom time was reallocated from teacher-centered content delivery to higher-order learning activities that emphasized music analysis, instrumental practice, collaborative discussion, creative music making, and musical problem-solving. By relocating lower-level cognitive tasks outside the classroom, instructional design created opportunities for students to engage more deeply in authentic musical learning during face-to-face instruction (Ng et al., 2022; Lv, 2023; Ozdamar and Yavuz-Konokman, 2024; Zhu et al., 2025).

Building upon this instructional foundation, learner-centered classroom practices transformed the role of students from passive recipients of knowledge into active participants in musical learning. Rather than focusing primarily on information transmission, classroom activities encouraged students to interpret musical works, participate in ensemble performance, exchange perspectives with peers, and apply theoretical knowledge through authentic musical practice. These active learning experiences promoted knowledge construction while strengthening students' musical understanding through continuous interaction with learning tasks and classroom activities (Fan, 2025; Perakaki, 2025; Ng et al., 2022).

The effectiveness of these learning environments was further strengthened through teacher facilitation and collaborative learning. Instead of functioning primarily as knowledge transmitters, teachers assumed facilitative roles by organizing collaborative inquiry, scaffolding classroom discussions, providing individualized guidance, and offering timely instructional support according to students' learning needs. At the same time, collaborative learning enabled students to exchange ideas, negotiate musical interpretations, provide peer feedback, and solve musical problems collectively. The interaction between teacher guidance and peer collaboration created supportive learning environments in which students assumed greater responsibility for learning while benefiting from continuous instructional and social support throughout the flipped learning process (Nethsinghe et al., 2023; Perakaki, 2025; Ozdamar and Yavuz-Konokman, 2024).

Another recurring feature across the reviewed studies was the integration of formative assessment as a mechanism for continuous learning regulation. Rather than serving solely as a means of evaluating achievement, formative assessment functioned as an ongoing instructional process incorporating instructor feedback, peer assessment, reflective self-evaluation, and continuous monitoring of learning progress. These assessment practices enabled students to identify misconceptions, refine musical understanding, improve practical performance, and regulate their own learning through iterative reflection and feedback. By embedding assessment within the instructional process, flipped music learning supported adaptive learning behaviors and sustained engagement beyond individual classroom activities (Doi, 2016; Zhu et al., 2025; Roig-Vila and Fabra-Brell, 2024).

Overall, the evidence suggests that effective flipped music learning environments emerge from the integration of mutually reinforcing pedagogical and contextual conditions rather than from any single instructional strategy. Structured instructional design provides the foundation for independent preparation; learner-centered classroom practices transform knowledge into authentic musical experiences; teacher facilitation and collaborative learning establish the instructional and social support necessary for active participation; and formative assessment promotes continuous reflection and learning regulation. Collectively, these interconnected factors establish a coherent pedagogical environment that supports meaningful engagement and creates the instructional conditions through which flipped music education contributes to students' subsequent learning experiences and broader educational outcomes, as synthesized in the following research questions.

### 3.2 RQ2: how do these factors influence students' engagement and learning experiences?

The synthesis of the reviewed studies indicates that the instructional and contextual factors identified in RQ1 influence students' engagement and learning experiences by fundamentally reshaping how learners participate in music learning. Rather than functioning as isolated instructional components, structured pre-class preparation, active in-class learning, teacher facilitation, collaborative learning, and technology-supported instruction interact to create learner-centered environments that encourage active participation, deeper learning, and sustained engagement (Doi, 2016; Perakaki, 2025; Zhu et al., 2025). Across the reviewed studies, these influences were consistently reflected in four interconnected dimensions of engagement: behavioral, cognitive, social, and emotional engagement.

Behavioral engagement was enhanced through the reallocation of learning activities across pre-class and in-class phases. Pre-class instructional videos and digital learning resources enabled students to acquire foundational musical knowledge independently, allowing classroom time to focus on music performance, discussion, creative activities, and problem-solving. This instructional reorganization encouraged students to participate more actively during lessons, assume greater responsibility for learning, and engage more consistently with musical tasks. Studies further suggest that students became increasingly autonomous in managing their learning process as flipped instruction promoted self-paced preparation and continuous participation (Brell and Roig-Vila, 2022; Roig-Vila and Fabra-Brell, 2024; Ozdamar and Yavuz-Konokman, 2024).

The reviewed evidence also demonstrates that flipped learning facilitates cognitive engagement by supporting active knowledge construction. Rather than relying primarily on teacher explanation, students were encouraged to analyze musical concepts, apply theoretical knowledge during instrumental practice and performance, solve authentic musical problems, and reflect on their own learning. The integration of digital technologies, including AI-assisted learning environments and music applications, further provided opportunities for individualized learning support and immediate feedback, enabling learners to connect theoretical understanding with practical musical application. These learning experiences promoted deeper conceptual understanding and higher-order thinking throughout the learning process (Ng et al., 2022; Lv, 2023; Sakin et al., 2024).

Social engagement emerged as another important mechanism through which flipped music learning enhanced students' learning experiences. The increased availability of classroom time enabled greater interaction among students through collaborative performance, peer discussion, ensemble practice, and shared musical inquiry. These collaborative learning opportunities encouraged learners to exchange ideas, negotiate musical interpretations, and provide constructive feedback, thereby strengthening interpersonal communication and collective knowledge construction. Such learner-centered interactions contributed to a more participatory and supportive classroom environment than traditional teacher-directed instruction (Nethsinghe et al., 2023; Ozdamar and Yavuz-Konokman, 2024).

In addition to behavioral, cognitive, and social engagement, the reviewed studies indicate that flipped music learning positively influences students' emotional engagement. Increased opportunities for successful participation, autonomy, creative expression, and supportive teacher guidance fostered greater learning motivation, confidence, and positive attitudes toward music education. Students frequently reported greater enjoyment of learning, stronger commitment to classroom activities, and higher levels of learning satisfaction when instructional activities were designed to promote active involvement and meaningful musical experiences. These motivational benefits were particularly evident in studies involving instrumental instruction and creative music-making activities (Fan, 2025; Yilmaz and Zahal, 2023; Ng et al., 2022).

Overall, the evidence suggests that students' engagement in flipped music education is shaped through the interaction of multiple pedagogical processes rather than by any single instructional strategy. Structured independent learning promotes behavioral participation; authentic musical inquiry facilitates cognitive engagement; collaborative classroom activities strengthen social interaction; and learner-centered environments foster positive emotional experiences. Together, these interconnected dimensions contribute to richer learning experiences and establish the educational mechanisms through which flipped music learning supports the broader cognitive, emotional, and wellbeing outcomes synthesized in RQ3.

### 3.3 RQ3: what cognitive, emotional, and wellbeing outcomes are associated with flipped music education?

The reviewed studies indicate that flipped music education is associated with a broad range of cognitive, emotional, and educational wellbeing outcomes that extend beyond improvements in musical knowledge alone. Across the included studies, these outcomes emerged through students' active participation in technology-enhanced learning environments, authentic musical practice, collaborative learning, and reflective learning processes, collectively contributing to both academic achievement and personal development.

At the cognitive level, flipped music education consistently enhanced students' conceptual understanding, musical competence, analytical thinking, problem-solving ability, and practical performance skills. Students demonstrated improved understanding of music theory by applying abstract concepts to authentic learning activities such as instrumental performance, ensemble practice, creative music making, and music analysis. The integration of digital technologies and AI-supported learning environments further facilitated individualized learning, enabling students to revisit instructional materials, receive immediate feedback, and develop deeper conceptual understanding through self-paced learning and repeated practice (Ng et al., 2022; Lv, 2023; Ozdamar and Yavuz-Konokman, 2024; Sakin et al., 2024; Zhu et al., 2025).

The reviewed evidence also demonstrates substantial emotional benefits associated with flipped music education. Students frequently reported higher levels of learning motivation, greater confidence in musical performance, and more positive attitudes toward music learning when they were provided with opportunities for autonomous preparation, active classroom participation, and creative musical expression. These learner-centered experiences enhanced students' enjoyment of learning while reducing anxiety associated with classroom participation, thereby fostering stronger emotional investment in the learning process. Such positive emotional responses were consistently observed across instrumental instruction, music theory, and collaborative music learning contexts (Fan, 2025; Yilmaz and Zahal, 2023; Doi, 2016).

Beyond cognitive and emotional development, flipped music education contributed to students' educational wellbeing by promoting greater learning satisfaction, stronger self-efficacy, increased learner autonomy, and sustained commitment to learning. Flexible access to digital learning resources, supportive teacher guidance, collaborative classroom environments, and opportunities for successful musical performance enabled students to develop greater confidence in their learning abilities while maintaining long-term engagement with music education. These findings suggest that flipped learning supports not only academic achievement but also broader educational wellbeing by creating learning environments in which students experience competence, autonomy, and meaningful participation (Brell and Roig-Vila, 2022; Perakaki, 2025; Roig-Vila and Fabra-Brell, 2024; Nethsinghe et al., 2023).

Overall, the reviewed evidence suggests that cognitive, emotional, and educational wellbeing outcomes are closely interconnected within flipped music education. Improvements in musical understanding and performance strengthen learners' confidence and motivation, while positive emotional experiences reinforce learning satisfaction, self-efficacy, and sustained engagement. Together, these complementary outcomes demonstrate that flipped music education contributes to students' holistic development by integrating cognitive growth, emotional development, and educational wellbeing, thereby providing the educational outcomes that underpin the explanatory mechanisms synthesized in RQ4.

### 3.4 RQ4: how can existing evidence be synthesized to explain the mechanisms through which flipped music education contributes to student development and wellbeing?

**Figure 2:**
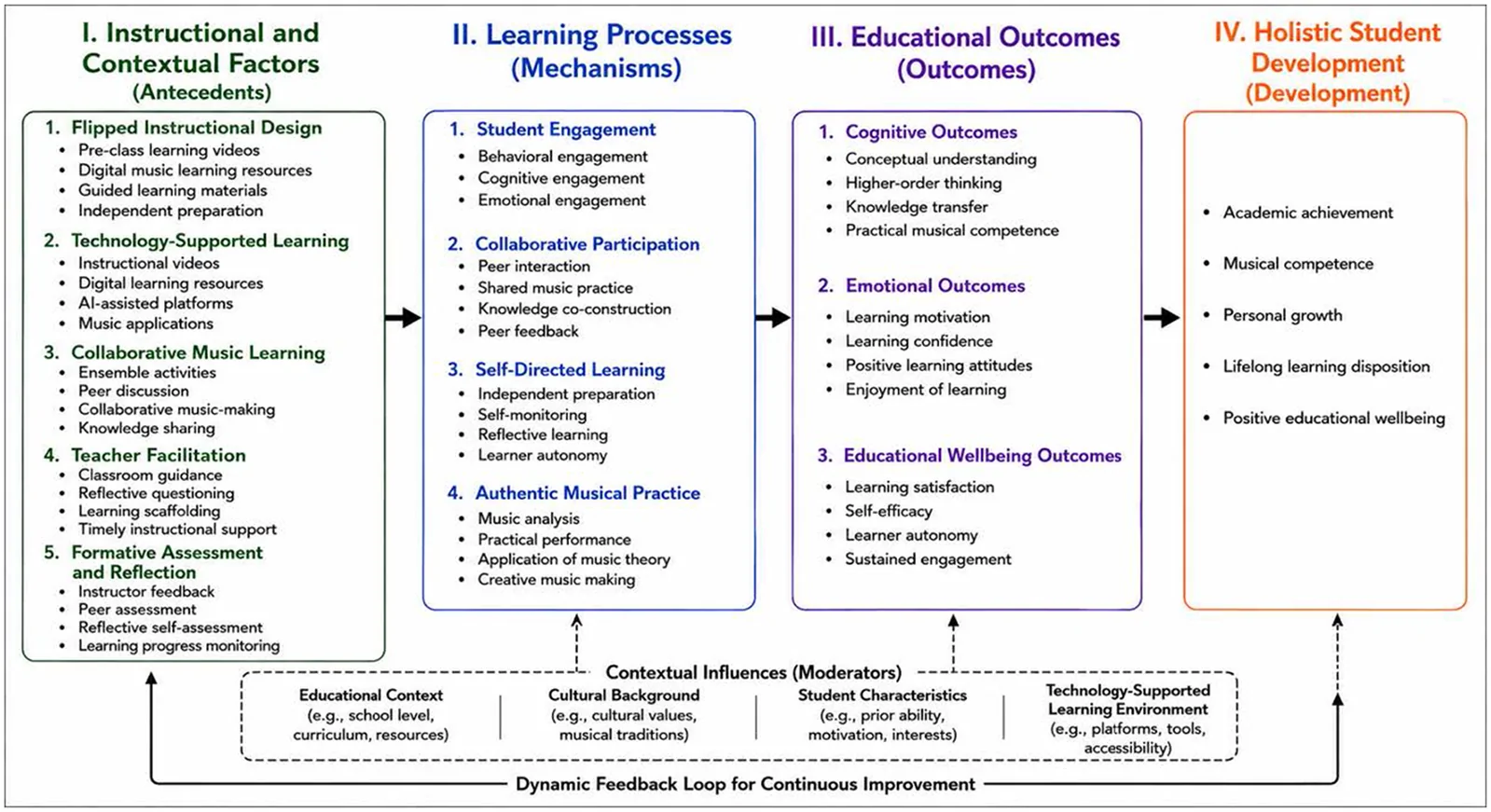
Conceptual framework of flipped music education.

The reviewed evidence was synthesized into the conceptual framework presented in Figure 2, which explains how flipped music education contributes to student development and educational wellbeing through the dynamic interaction of instructional conditions, learning processes, and developmental outcomes. Rather than representing isolated educational components, these dimensions operate as an integrated pedagogical system in which instructional and contextual factors facilitate learner engagement, engagement drives meaningful learning experiences, and sustained participation ultimately contributes to students' holistic development.

The framework proposes that instructional and contextual factors constitute the foundation of effective flipped music education. Across the reviewed studies, structured instructional design, technology-supported learning resources, collaborative music learning, teacher facilitation, and formative assessment collectively establish learner-centered environments that encourage students to prepare independently before class while actively participating during classroom learning. These pedagogical conditions provide the structural support necessary for students to engage with musical knowledge through authentic learning experiences rather than passive content acquisition (Doi, 2016; Ng et al., 2022; Lv, 2023; Ozdamar and Yavuz-Konokman, 2024; Perakaki, 2025; Zhu et al., 2025).

Building upon these instructional conditions, the framework identifies four complementary learning processes that explain how flipped music education promotes learning. Student engagement encourages behavioral, cognitive, and emotional involvement in learning; collaborative participation enables peer interaction and shared knowledge construction; self-directed learning strengthens learner autonomy through independent preparation and reflective learning; and authentic musical practice connects theoretical knowledge with practical performance, creative music making, and musical interpretation. Together, these learner-centered processes function as the principal mechanisms through which instructional conditions are translated into meaningful educational experiences (Fan, 2025; Ng et al., 2022; Nethsinghe et al., 2023; Sakin et al., 2024; Yilmaz and Zahal, 2023).

The framework further illustrates that these learning processes generate multiple and mutually reinforcing educational outcomes. Sustained engagement in flipped music learning contributes simultaneously to cognitive development, including improved conceptual understanding, higher-order thinking, and musical competence; emotional development, reflected in increased motivation, confidence, enjoyment, and positive learning attitudes; and educational wellbeing, characterized by greater learning satisfaction, self-efficacy, learner autonomy, and sustained engagement. Rather than occurring independently, these outcomes reinforce one another and collectively support students' broader educational development (Brell and Roig-Vila, 2022; Fan, 2025; Roig-Vila and Fabra-Brell, 2024; Zhu et al., 2025).

Overall, the proposed framework synthesizes the evidence by demonstrating a sequential explanatory pathway in which instructional and contextual factors establish supportive learning environments, learner-centered learning processes operate as the core developmental mechanisms, and cognitive, emotional, and educational wellbeing outcomes collectively contribute to holistic student development. The framework therefore provides an integrated explanation of how flipped music education promotes meaningful learning across diverse music education contexts and offers a theoretical foundation for future instructional design and empirical research.

## 4 Discussion

### 4.1 Addressing existing research gaps

Although flipped learning has been widely adopted in music education, existing research has primarily concentrated on evaluating instructional effectiveness, technological implementation, and improvements in academic performance. Consequently, current knowledge remains fragmented, with limited understanding of the mechanisms through which flipped music education promotes broader student development. Most empirical studies have investigated individual instructional components, such as pre-class videos, classroom activities, or digital technologies, without explaining how these elements collectively influence students' learning processes and developmental outcomes.

This review addresses these gaps by adopting a mechanism-oriented perspective. Rather than asking whether flipped music education is effective, the review explains how instructional and contextual factors interact with learner-centered learning processes to produce cognitive, emotional, and educational wellbeing outcomes. By synthesizing evidence across diverse educational settings, the review integrates previously disconnected findings into a coherent explanatory framework, thereby extending current understanding of flipped music education beyond instructional effectiveness toward educational development.

### 4.2 Advancing a mechanism-oriented understanding of flipped music education

The principal contribution of this review lies in advancing a mechanism-oriented understanding of flipped music education. Unlike previous research that has primarily described flipped learning as a teaching strategy or technological innovation, this review conceptualizes flipped music education as a dynamic pedagogical system in which instructional conditions, learning processes, and developmental outcomes operate as interconnected components.

The synthesis demonstrates that instructional design alone does not directly generate positive educational outcomes. Instead, technology-supported learning, teacher facilitation, collaborative learning, and formative assessment establish supportive learning environments that encourage learner engagement, collaborative participation, self-directed learning, and authentic musical practice. These learner-centered processes function as the mechanisms through which instructional conditions are translated into meaningful educational experiences.

Based on this evidence, the review proposes an explanatory conceptual framework that connects instructional and contextual factors, learner-centered learning processes, educational outcomes, and holistic student development within a single theoretical model. This framework extends previous literature in three important ways. First, it shifts the focus from evaluating instructional effectiveness to explaining the processes underlying educational change. Second, it identifies learner engagement and collaborative participation as central mechanisms connecting pedagogy with development. Third, it integrates cognitive development, emotional development, and educational wellbeing within one explanatory structure, demonstrating that these outcomes reinforce one another rather than occurring independently. Collectively, these contributions provide a stronger theoretical foundation for future research on flipped learning in music education.

### 4.3 Practical implications

The findings suggest that effective flipped music education requires more than the adoption of digital technologies or pre-class instructional videos. Successful implementation depends on designing learner-centered environments that combine independent preparation, collaborative inquiry, authentic musical practice, and continuous formative feedback. These instructional elements should be viewed as complementary rather than isolated pedagogical strategies.

The review also highlights the changing role of music educators. Within flipped learning environments, teachers function as facilitators who scaffold learning, promote collaboration, provide individualized feedback, and support students' self-directed learning. Accordingly, professional development programms should strengthen teachers' pedagogical competencies in addition to technological skills. Furthermore, curriculum designers should recognize educational wellbeing as an important educational objective alongside musical competence, ensuring that music learning environments support students' motivation, confidence, learner autonomy, and long-term engagement.

### 4.4 Limitations and future research

Several limitations should be considered. The reviewed studies demonstrated considerable variation in research design, intervention duration, participant characteristics, and outcome measures, limiting direct comparison across studies. Only English-language publications were included to ensure consistent interpretation of study methods and findings during the review process. Although AI-assisted translation has improved accessibility to non-English literature, this criterion may have introduced language bias and is acknowledged as a limitation of the review. The included studies were conducted across diverse countries, educational systems, and participant populations, resulting in considerable contextual heterogeneity. Although this diversity enhances the broader relevance of the review, it may also limit the generalizability of individual findings because the implementation and effectiveness of flipped music education are likely to be influenced by local educational contexts, cultural factors, and learner characteristics. Future research should validate the proposed conceptual framework through cross-cultural comparative studies and examine how contextual variables moderate the relationships identified in this review. In addition, most evidence was derived from short-term interventions conducted in higher education, restricting understanding of long-term developmental effects and reducing generalizability across educational levels and cultural contexts.

Future research should empirically validate the proposed conceptual framework using longitudinal, mixed-method, and cross-cultural research designs. Studies employing structural equation modeling, mediation analysis, or learning analytics may further examine the relationships among instructional conditions, learner-centered learning processes, and educational outcomes. Future investigations should also explore how contextual factors, learner characteristics, and emerging technologies, including artificial intelligence, influence the effectiveness of flipped music education. Such research would strengthen the evidence base and further advance mechanism-oriented theory development within music education.

## 5 Conclusion

This systematic review synthesized evidence from 12 empirical studies to advance understanding of flipped music education from a mechanism-oriented perspective. Rather than focusing solely on instructional effectiveness, the review integrated existing evidence to explain how instructional and contextual factors, learner-centered learning processes, and educational outcomes interact to support holistic student development. The principal contribution of this review is the development of an explanatory conceptual framework that clarifies how flipped music education facilitates cognitive, emotional, and educational wellbeing outcomes by linking instructional conditions with learner-centered learning processes. By shifting attention from whether flipped music education is effective to how and why it contributes to student development, the proposed framework addresses an important gap in the existing literature and extends current theoretical understanding of flipped music education. In addition, the review highlights the importance of designing learner-centered music learning environments that promote meaningful participation, collaboration, reflection, and authentic musical practice alongside technology-supported instruction. Although the proposed framework requires further validation across different educational contexts, it provides an evidence-based foundation for future research, curriculum development, and instructional practice in flipped music education.
