# Correction: Mechanisms and outcomes of flipped music education: a conceptual framework linking self-directed learning, collaboration, and student wellbeing

**DOI:** 10.3389/fpsyg.2026.1947747

**Published:** 2026-09-15

**Authors:** Qisen Zhu, Jinglong Li

**Affiliations:** 1 Faculty of Education, University Kebangsaan Malaysia, Selangor, Malaysia; 2 Industrial Design College, LuXun Academy of Fine Arts, Shenyang, China

**Keywords:** collaborative learning, emotional regulation, flipped music education, learning engagement, self-directed learning, student wellbeing

Affiliation “Industrial Design College, LuXun Academy of Fine Arts, Shenyang, China” was omitted from the paper. This affiliation has now been added for author Jinglong Li.

Affiliation “Department of Industrial Design, Faculty of Design and Architecture, University Putra Malaysia, Serdang, Malaysia” was erroneously included in the paper. This affiliation has now been removed for author Jinglong Li.

Author Qisen Zhu was erroneously included as corresponding author. The correct corresponding author(s) is Jinglong Li.

The original version of this article has been updated.

